# High sensitivity of room-temperature terahertz photodetector based on silicon

**DOI:** 10.1016/j.isci.2022.105217

**Published:** 2022-09-26

**Authors:** Qinxi Qiu, Wanli Ma, Jingbo Li, Lin Jiang, Wangchen Mao, Xuehui Lu, Niangjuan Yao, Yi Shi, Zhiming Huang

**Affiliations:** 1 State Key Laboratory of Infrared Physics, Shanghai Institute of Technical Physics, Chinese Academy of Sciences, 500 Yu Tian Road, Shanghai 200083, P. R. China; 2 University of Chinese Academy of Sciences, 19 Yu Quan Road, Beijing 100049, P. R. China; 3 Key Laboratory of Polar Materials and Devices, Department of Electronic Engineering East China Normal University, 500 Dong Chuan Road, Shanghai 200241, P. R. China; 4 Key Laboratory of Space Active Opto-Electronics Technology, Shanghai Institute of Technical Physics, Chinese Academy of Sciences, 500 Yu Tian Road, Shanghai 200083, P. R. China; 5 Hangzhou Institute for Advanced Study, University of Chinese Academy of Sciences, 1 Sub-Lane Xiangshan, Hangzhou 310024, P. R. China; 6 Institute of Optoelectronics, Fudan University, 2005 Song Hu Road, Shanghai 200438, P. R. China

**Keywords:** Radiation physics, engineering, devices

## Abstract

Silicon (Si) is the most important semiconductor material broadly used in both electronics and optoelectronics. However, the performance of Si-based room temperature detectors is far below the requirements for direct detection in the terahertz (THz) band, a very promising electromagnetic band for the next-generation technology. Here, we report a high sensitivity of room temperature THz photodetector utilizing the electromagnetic induced well mechanism with an SOI-based structure for easy integration. The detector achieves a responsivity of 122 kV W^−1^, noise equivalent power (NEP) of 0.16 pW Hz^−1/2^, and a fast response of 1.29 μs at room temperature. The acquired NEP of the detector is ∼2 orders lower in magnitude than that of other types of Si-based detectors. Our results pave the way to realize Si-based THz focal plane arrays, which can be used in a wide range of applications, such as medical diagnosis, remote sensing, and security inspection.

## Highlights

• The SOI detector is based on the electromagnetic induction well mechanism
• The SOI detectors achieved highly sensitive detection at terahertz room temperature
• The SOI terahertz detector has a fast response capability

## Graphical abstract

**Figure:**
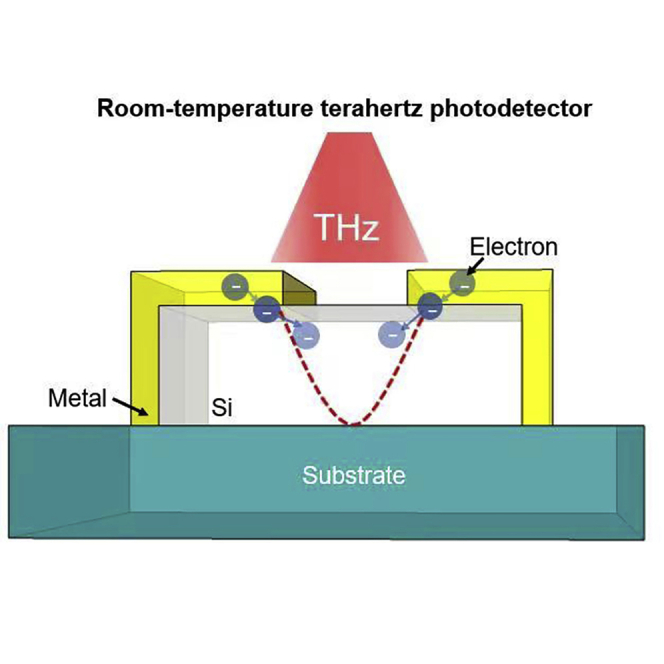

## Introduction

Terahertz (THz) wave is an electromagnetic wave between infrared and microwave with a frequency of 0.1-10 THz (wavelength of 30-3000 μm). Owing to its excellent characteristics, it shows great potential in communication, non-destructive testing, biological, security, imaging, and other fields (Kim et al., 2020; Suzuki et al., 2021; Shang et al., 2021; Takida et al., 2021; Bai et al., 2019; Liu et al., 2021). THz detector as the signal collector is one of the key factors in the application of terahertz technology. The existing terahertz detectors can be divided into cooled and uncooled detectors. Cooled detectors have high sensitivity, but require low-temperature cooling and complex structure. Uncooled terahertz detectors include Golay, microbolometer, field-effect transistors (FETs), etc. Sensitive detectors are available based on bulk materials, such as InGaAs, and Si, as well as two-dimensional materials, such as carbon nanotubes, etc (Sizov, 2018; Rogalski et al., 2019; Jin et al., 2021). However, these detectors suffer from slow corresponding speed, low sensitivity, and instability. High performance of low power THz detector with high sensitivity, fast response, broadband response and can be integrated on-chip is always pursued.

Si is the most important semiconductor with mature, reliable, inexpensive production and the ability to grow over a large area. Si has a cut-off wavelength of 1100 nm, making it difficult to achieve high-performance terahertz detection, but also reducing the effect of the infrared background on its performance. Much effort has been paid to developing Si-based THz detectors. Among them, Silicon on Insulator (SOI) with a tri-layer structure of Si/SiO_2_/Si is the most promising silicon technology today (Rudenko et al., 2020). SOI has been used in the manufacture of integrated circuits and key electronic components for many mainstream electronic applications, radio frequency front-end modules, chemical and biological integrated sensors, optical waveguides, and photonic circuits (Lee et al., 2020; Tang et al., 2021; Khozeymeh et al., 2018; Zhang et al., 2020; Aalto et al., 2019). Compared with bulk Si, SOI has the advantages of faster operating speed, lower power consumption, and higher operating temperature (Tibenszky et al., 2020; Dao et al., 2021; Ghosh and Iizuka, 2021). Therefore, THz detectors based on SOI have arisen abundant research interest. The reported typical structure of SOI THz detectors is field-effect transistors based on plasma wave theory initiated by Dyakonov and Shur (Dyakonov and Shur (1993). The noise equivalent power (NEP) for Junctionless FET manufactured on SOI wafers was estimated to be 460 pW Hz^−1/2^ (Marczewski et al., 2015). T-Channel junctionless FET was fabricated on SOI wafers to achieve a maximum signal-to-noise ratio of 84 dB at 0.22–0.33 THz (Zaborowski et al., 2019). The nFET detector in an advanced 22 nm SOI complementary metal-oxide-semiconductor (CMOS) technology was achieved 22.65 pW Hz^−1/2^ at 0.855 THz (Jain et al., 2018). Nevertheless, the NEP of these detectors was larger than 10 pW Hz^−1/2^ at room temperature. More importantly, the calculated theoretical NEP limits can only be larger than 1 pW Hz^−1/2^ for FET structure, for example, a FET with a channel length of 90 nm and width of 1 μm, its lowest theoretical NEP is about 3 pW Hz^−1/2^ at room temperature.^[23]^

However, the NEP of the detector is required to be < 0.01–0.1 pW Hz^−1/2^ for THz direct detection passive imaging systems (Golenkov and Sizov, 2016). Hence, the NEP of those SOI-based detectors cannot meet the requirements in real applications, there is an urgent need to develop high sensitivity of SOI-based THz photodetectors.

Here, we use SOI to fabricate THz room temperature photodetectors with different mesa sizes, based on the THz detection mechanism of electromagnetic induced wells (EIWs). This mechanism enables high sensitivity detection of THz at room temperature, and the predicted theoretical value of NEP for Si detectors based on this mechanism is ∼1 fW Hz^−1/2^ order of magnitude (When the resistivity of silicon is 0.07 Ω cm and the length, width, and thickness of the detector mesa are 2 μm, 0.5 μm, and 0.5 μm, respectively, the theoretical responsivity of the detector is 10^6^ V/W and the theoretical noise of the detector is in the order of nV/Hz^1/2^.), which is much better than the estimated value of the detectors based on other mechanisms and can meet the needs of THz direct detection passive imaging systems. The realized detector shows NEP of as low as 0.16 pW Hz^−1/2^ and a fast response of 1.29 μs at room temperature, which is expected to be compatible with integrated circuits to manufacture a terahertz focal plane array.

## Results and discussion

**Figure 1:**
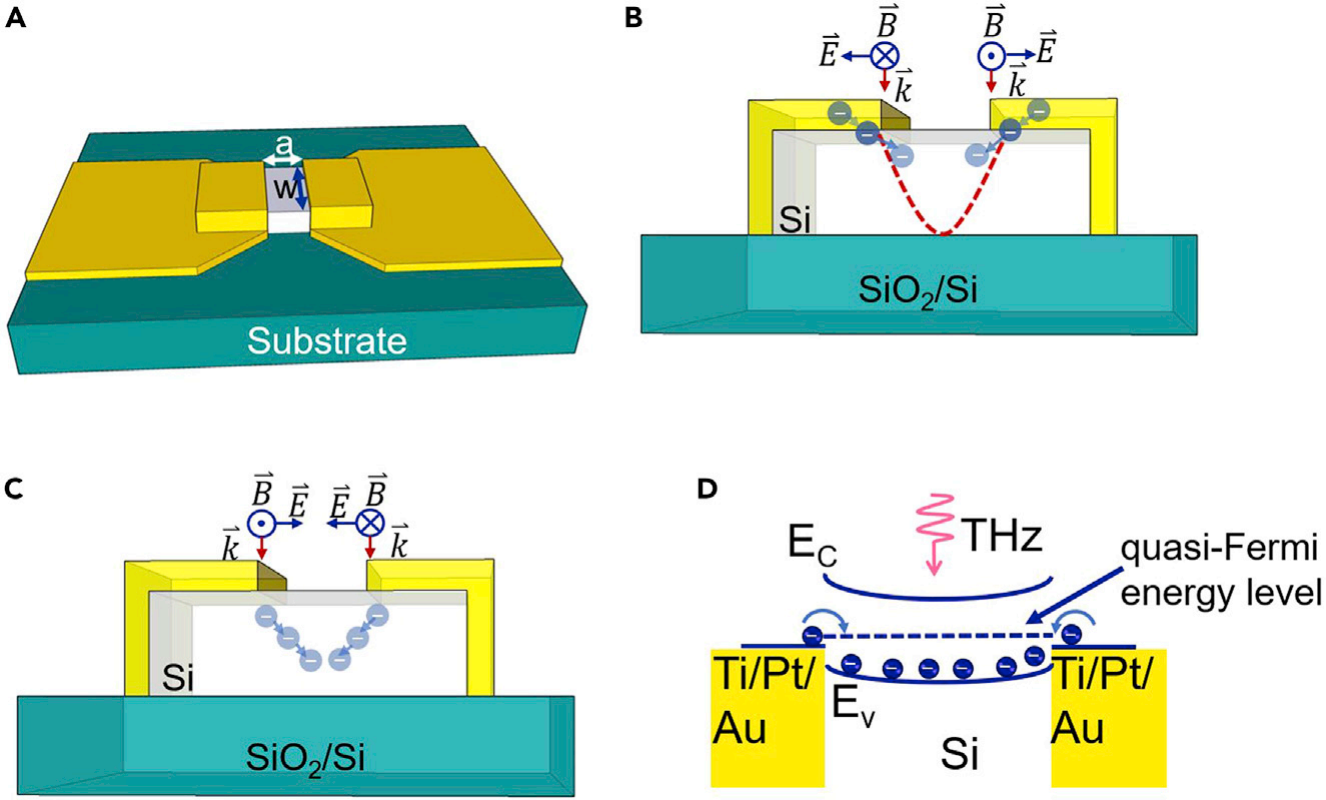
Schematic diagram of the photoelectric effect of the Si terahertz detector (A) Schematic diagram of the Si terahertz detector, where *a* is the gap length, *w* is the mesa width, and *d* is the thickness of the SOI device layer. (B) The antisymmetric electric field generates an electromagnetic induced wells in Si, and the electrons in the metal are injected into the Si by Lorentz force. (C) The symmetrical electric field creates a potential barrier, and the previously injected electrons still drift to the center of the material in slow motion under the action of the Lorentz force. (D) The injected electrons in P-type Si recombine with holes, causing the quasi-Fermi energy level of Si to shift upward.

Huang et al. proposed a photoelectric effect mechanism for THz detection at room temperature, which uses THz radiation to inject electrons from the metals on both sides of a metal-semiconductor-metal (MSM) structure with a subwavelength gap into the semiconductor. Figure 1A shows a schematic diagram of a metal-Si-metal structure with a subwavelength gap based on SOI. If the gap length *a* is greater than the shielding length of electrons but much less than the incident wavelength, the antisymmetric electric field of terahertz radiation can generate an electromagnetic induced well in the semiconductor when a terahertz wave irradiates the structure. Owing to the large difference in electron concentration between the metal (ca. 5.89×10^22^ cm^−3^) and the Si (ca. 9×10^18^ cm^−3^), the electrons in the metal are injected into Si from the two top metal layers driven by Lorentz force and are trapped by the potential well, as shown in Figure 1B. In the following half period, an induced potential barrier is generated, and the injected electrons in Si decelerated but further move forward under the Lorentz force (Figure 1C). As a result, the injected electrons do not return to the two metallic electrodes, but accumulate in Si, thereby reducing its resistance. The injected electrons in P-type Si recombine with holes, causing the quasi-Fermi energy level of Si to shift up and the conductivity to drop, thus allowing the detection of terahertz radiation (Figure 1D). If the potential barrier is formed first in the half period, electrons will still be injected during the half period when the potential well is formed, because the carrier concentration of the semiconductor is very different from that of the metal (Huang et al., 2014, 2016).

According to the D'Alembert equation (Feynman et al., 1964) and the transport process of carriers, we can calculate the concentration of injected electrons in the semiconductor:

(Equation 1) $$\Delta n=\frac{4\varepsilon_{0}PY\left(a\right)}{\pi^{3}q^{2}cd\sqrt{\varepsilon_{r}}},$$

where *ε*_*0*_ is the permittivity in vacuum, $Y\left(a\right)=a\eta \sqrt{{\left(\frac{\pi }{a}\right)}^{2}-k_{0}^{2}}\times \left[1-\text{exp}(-d\sqrt{\varepsilon_{r}}\sqrt{{\left(\frac{\pi }{a}\right)}^{2}-k_{0}^{2}})\right]$, $\eta =\left|\frac{\varepsilon \left(\omega \right)\sqrt{k_{0}^{2}-{\left(\frac{\pi }{a}\right)}^{2}}}{\sqrt{\varepsilon \left(\omega \right)k_{0}^{2}-{\left(\frac{\pi }{a}\right)}^{2}}}\right|$ (*ε(ω)* is the relative permittivity of the metal) is the electric field enhancement factor in the gap, *P* is the power of incident light, *a* is the gap length, *q* is the unit electric charge, *c* is the speed of light, *d* is the thickness of Si, *ε*_*r*_ is the relative permittivity of Si, and *k*_*0*_ is the wave vector of light in vacuum. It can be seen from Equation 1 that Δn is proportional to the power of incident light and is a function of Y(a) and mesa thickness *d*.

The variation of silicon electron concentration leads to the change in resistance, ignoring the variation of silicon carrier mobility, the variation of detector resistance can be described as:

(Equation 2) $$\Delta R=\frac{-\left(\mu_{e}\Delta n+\mu_{h}\Delta h\right)}{q{\left(\mu_{e}n+\mu_{h}h\right)}^{2}}\cdot \frac{a}{dw},$$

where *μ*_*e*_ and *μ*_*h*_ are electron and hole mobility, respectively, and *n* and *h* are the electrons and holes concentration of Si, respectively, *w* is the gap width. The signal voltage across the detector is:

(Equation 3) $$V_{ph}=I_{b}\Delta R=\frac{4\varepsilon_{0}I_{b}R_{a}Y\left(a\right)P}{\pi^{3}q^{2}dcn\sqrt{\varepsilon_{r}}},$$

where *I*_*b*_ is the bias current, and *R*_*a*_ is the resistance of the detector. As shown in Equation 3, the signal voltage of the detector is related to the resistance of the detector and Y(a), so the signal voltage is related to the length *a*, width *w*, and thickness *d* as follows.

(Equation 4) $$V_{ph}\propto \frac{aY\left(a\right)}{d^{2}w}$$

It can be seen from Figure S3 (See supplemental information S3) that the smaller *a* is, the larger Y(a) is. Therefore, the smaller the *a*, *d*, and *w* in a certain range according to the equation, the higher the photovoltage of the detector. By decreasing the thickness d of the top mesa layer, the photovoltage of the detector becomes better.

**Figure 2:**
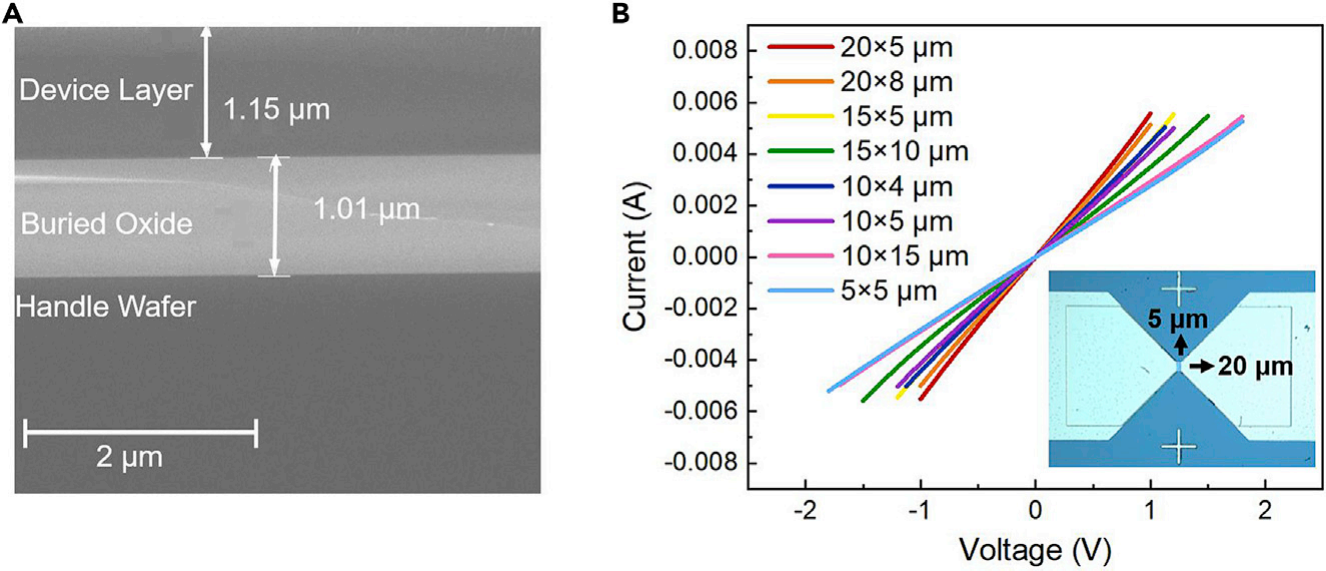
SOI structure and electrical characteristics of the detectors (A) SEM cross-section of SOI material. (B) Dark I-V curves of Si photodetectors with different mesa sizes. Inset: Optical photograph of Si photodetector with MSM structure.

As shown in Figure 2A, the Si THz detector was fabricated using SOI material with a tri-layer structure of Si/SiO_2_/Si, in which the thickness of the detector layer p-type Si is 1.15 μm and resistivity is 0.01–0.05 Ω cm, the buried oxide layer is SiO_2_ with a thickness of about 1 μm, the Si of the handle layer is of a thickness of 510 μm and resistivity is greater than 10000 Ω cm. We fabricated two structures of detectors, one with a mesa and the other is a planar detector without a mesa. See the experimental section for the specific procedure of the two structures of detectors. Both the planar detector and the mesa detector had the same antenna size. The dark current-voltage (I-V) curves for detectors with different mesa sizes (Figure 2B) and the planar detector (See supplemental information S4) show that the detectors have formed ohmic contacts.

**Figure 3:**
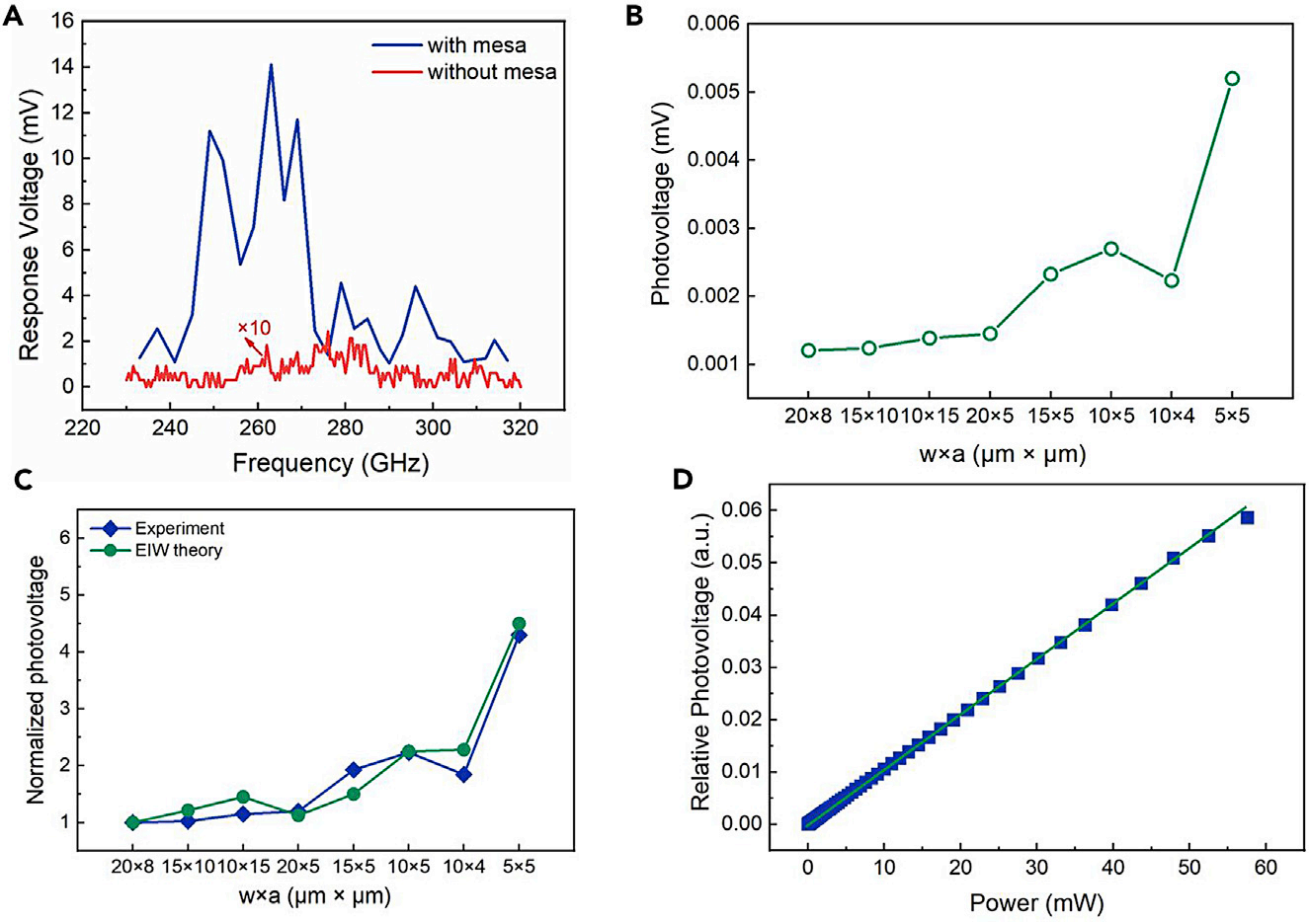
Photoelectric characteristics of Si terahertz detectors (A) The response voltages of the SOI terahertz detector with a mesa and the planar SOI terahertz detector multiplied by a factor of 10 at 0.23–0.32 THz. (B) Photovoltage of detectors with different mesa widths and gap lengths at 0.263 THz. (C) The photovoltage of the detector with a mesa width of 20 μm and a gap length of 8 μm is set to 1. The ratio of photovoltage of detectors with different mesa sizes is roughly consistent with EIW theory. (D) Photovoltage of the Si detector as a function of source powers.

The room temperature response performance of 0.23–0.32 THz was measured for both types of detectors at a bias current of 5 mA. From Figure 3A it can be seen that the planar detector has no observable response only noise at 0.23–0.32 THz, while the detector with mesa has an obvious response voltage. As the planar detector and mesa detectors have the same size of antenna and irradiation spot, the antenna and irradiation spot have the same effect on both types of the detectors, such as the impedance, gain of the antenna, and power density. Therefore, the difference in response voltage between the two types of detectors at 0.23–0.32 THz is only attributed to the presence or absence of the mesa. This is because the top and both sides of the mesa detectors are covered with gold, which prevents electromagnetic wave leakage from both ends and enables large optical gain. The planar detector will lead to electromagnetic wave leakage, so the EIW effect cannot be applied to achieve terahertz detection. Moreover, the Si terahertz detectors with different mesa sizes have a maximal response voltage of around 0.263 THz (see supplemental information S5), the maximal response voltage of the detectors was chosen for comparison. As shown in Figure 3B, the photovoltage of the Si terahertz detector gradually increases as the detector mesa size decreases, indicating that the photovoltage of the detector is related to mesa size, which is predicted exactly by the theory of EIWs (Figure 3C). Figure 3D plots the relative photovoltage as a function of incident power P at the bias current I_b_ = 5 mA. It shows that the photovoltage of the detectors was linear with the incident power, which is consistent with Equation 3.

**Figure 4:**
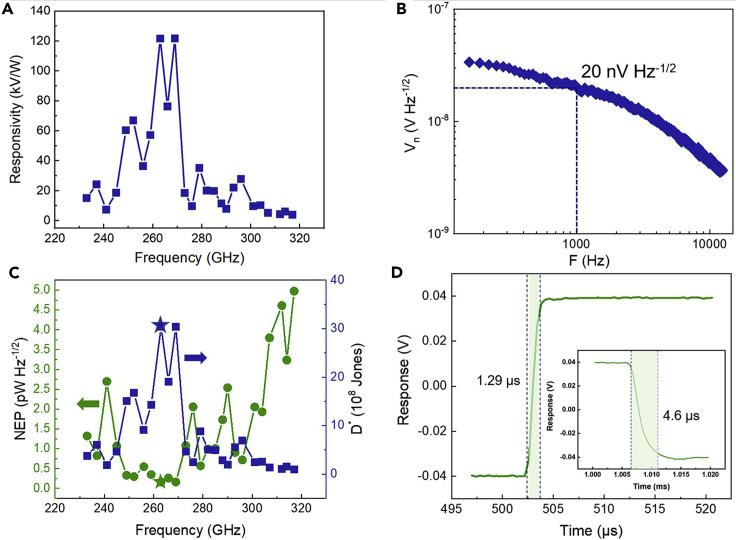
Photoelectric characteristics of Si terahertz detector with both mesa width and gap length of 5 μm (A) Responsivity of the Si detector at a frequency from 0.23 to 0.32 THz at a bias current of 5 mA. The peak responsivity located at 0.263 THz reaches 122 kV W^−1^. (B) Noise spectrum of the detector at 5 mA bias current. (C) NEP and D^∗^ of the detector at 0.23–0.32 THz. The lowest NEP and the largest D^∗^ at 0.263 THz are 0.16 pW Hz^−1/2^ and 3.04×10^9^ Jones, respectively. (D) The rise time of the detector response is 1.29 μs and a fall time of 4.6 μs (inset) at room temperature.

Based on the above discussion, we further calculate the performance of the Si detector. The responsivity can be derived by dividing the photovoltage by the power received by the detector. As mentioned above, the photovoltage of the detector is related to the mesa size, so the area of the detector is the area of the mesa when calculating the responsivity. Figure 4A shows the excellent responsivity of the detector for both the mesa width and gap length of 5 μm in the 0.23–0.32 THz. The peak responsivity is 122 kV W^−1^, located near 0.263 THz. Such high responsivity can be compared with commercial Golay cells (100 kV W^−1^). NEP is an important figure of merit for evaluating detector performance, defined as the lowest detectable power per unit bandwidth. It can be expressed as $NEP=V_{n}/R_{v}$, where *V*_*n*_ is the noise voltage, *R*_*v*_ is the responsivity of the detector. We measured the noise voltage of the detector is 20 nV Hz^−1/2^ at 1 kHz at 5 mA bias current using a spectrum analyzer (Figure 4B). Figure 4C shows the NEP of the detector at 0.23–0.32 THz. It can be seen that the Si detector has a pretty low NEP of 0.16 pW Hz^−1/2^ near 0.263 THz, which is lower than most reported THz photodetectors, as shown in Table 1. It is two orders of magnitude lower than Si FET/MOSFET and meets the needs of THz direct detection passive imaging systems. Although our experiments did not reach the theoretical expectation, the performance of the detector can be further improved by changing the carrier concentration of Si, improving the process to increase the response voltage of the detector and reduce the noise of the detector. Specific detectivity is a key index to evaluate detector performance and can be extracted from $D^{*}=\frac{\sqrt{A}}{NEP}$ (*A* is the detector area). According to the calculation, the maximum value of *D∗* is 3.04×10^9^ Jones (cm Hz^1/2^ W^−1^) near 0.263 THz.

**Table 1:** Comparison of uncooled terahertz wave detectors

Detector	Responsivity	NEP (W Hz^−1/2^)	Frequency (THz)	Response time (s)
Golay (Golay commercial)	100 kV/W	10^−10^-10^−9^	0.02–20	10^–2^
Pyroelectric (Kuznetsov et al., 2016)	100 kV/W	10^–9^	<30	10^–1^
Microbolometer (VO_x_, α-Si, Nb_5_N_6_, Ti) (Jiang et al., 2018; Rogalski, 2019; Simoens, and Meilhan, 2014)	4.97 kV/W	10^–11^	<3	10^−6^-10^−2^
Schottky-barrier diode (VDI ZBDs commercial)	1700 V/W	10^–12^	<0.4	10^–9^
Si FET/Si MOSFET (Khan et al., 2018; Deng et al., 2018)	4000 V/W	10^−11^-10^−10^	<0.7	–
AlGaN/GaN (Hou et al., 2017)	15.5 kV/W	5.8×10^−13^	0.14	–
Graphene (Castilla et al., 2019)	105 V/W	8 × 10^−11^	1.8–4.25	<3 × 10^−8^
EuBiSe_3_ Single Crystal (Wang et al., 2019)	0.69 V/W	1.1 × 10^−9^	1.84	9.7 × 10^−2^
CH_3_NH_3_PbI_3_ Perovskite (Li et al., 2020)	271 mA/W	3 × 10^−10^	2.52	1.26 × 10^−7^
Si (5 × 5 μm) (this work)	122 kV/W	1.6 × 10^−13^	0.23–0.32	1.29 × 10^−6^

For applications that require fast imaging, such as medical imaging, security inspection, and remote sensing detection, response time is another key performance of the detector. The rise and fall times were derived from the waveform. The rise time (defined here as the time required from 10% to 90% of the maximum photovoltage) is 1.29 μs, and the fall time (defined similarly as the time taken to from 90% to 10% of the maximum photovoltage) is 4.6 μs (Figure 4D), which is much faster than commercial millisecond response Golay and bolometer detectors.

**Figure 5:**
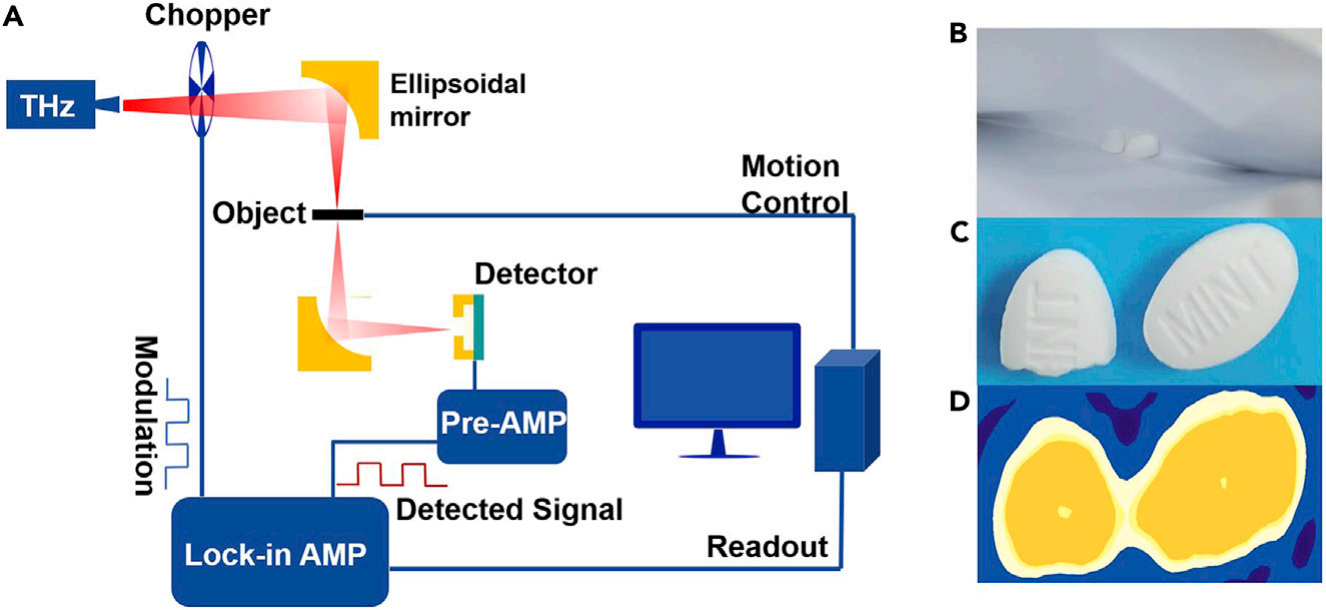
Room-temperature THz imaging using Si terahertz detector (A) Schematic diagram of the terahertz imaging system. The modulated terahertz source (modulate frequency 1 kHz) is focused on the detector through a series of lenses. During the measurement, the object is moved by the step controller. (B) A half and a whole mint in the envelope. (C) Optical pictures of half and whole mints. (D) Transmission image of the mint at 0.263 THz.

The photon energy of the terahertz wave is so small that it does not cause harm to humans, and it is easily transmitted through most non-metallic and non-polar mediums, so that the system can be used for security inspections, food quality inspections, cancer detection, etc. We performed room temperature THz imaging with the test system in Figure 5A. The source of the THz imaging system is modulated 0.263THz, which is focused on the detector through a series of lenses. A half and a whole piece of mint placed in the envelope between the THz source and the detector is selected as the target for THz imaging. Through the movement of the two-dimensional platform, the signal of the THz penetrating object is gradually collected through the preamplifier and the lock-in amplifier. Figure 5B shows the imaging results of a half mint and a whole mint inside the envelope. It can be seen that the terahertz wave can detect objects covered by article. Therefore, EIW-based Si detectors are expected to have better application prospects in THz imaging.

### Conclusions

In conclusion, we have manufactured a series of SOI-based photodetectors with different mesa sizes based on the detection mechanism of electromagnetic induced wells. Our experiments have demonstrated that the smaller the mesa width and gap length, the better performance of the detector, which is consistent with the EIW mechanism. We have successfully achieved the detector with a fast and sensitive response in the frequency of 0.23–0.32 THz at room temperature. The detector has reached a maximal responsivity of 122 kV W^−1^ at 0.263 THz. Importantly, the detector achieves low NEP (0.16 pW Hz^−1/2^), which is ∼2 orders of magnitude lower than the reported Si THz detectors and reaches the requirement of direct THz detection. The performance of the detector can be further improved by adjusting the electrical properties of the silicon. This high-performance Si detector is expected to be compatible with the mature CMOS integrated circuits technology to realize THz focal plane arrays and has great potential for applications in the THz imaging system.

### Limitations of the study

The influence of the thickness of the semiconductor material on the EIW mechanism and the limit of the material thickness can be further investigated.

## Data availability statement

All data generated or analyzed during this study are included in this article.

## STAR★Methods

### Key resources table

**Table:** 

REAGENT or RESOURCE	SOURCE	IDENTIFIER
**Chemicals, peptides, and recombinant proteins**		

SOI	Ultrasil LLC	Lot# UD-13884
Titanium(Ti) material sputtering Target	ACI Alloys	CAS#7440-32-6
Platinum (Pt) material sputtering Target	ACI Alloys	CAS# 7440-06-4
Gold (Au) material sputtering Target	ACI Alloys	CAS# 7440-57-5

**Software and algorithms**		

Origin 8	Origin Lab	http://www.originlab.com/

### Resource availability

#### Lead contact

Further information and requests for resources should be directed to and will be fulfilled by lead contact, Prof. Zhiming Huang (zmhuang@mail.sitp.ac.cn).

#### Material availability

This data not generate new unique reagents.

### Experimental model and subject details

This study does not use experimental methods typical in life sciences.

### Method details

#### Material and device manufacture

The Si photodetector was fabricated using SOI material with a tri-layer structure of Si/SiO_2_/Si, in which the p-type Si of the device layer is of a thickness of 1.15 μm and resistivity of 0.01–0.05 Ω cm, SiO_2_ of the buried oxide layer is of a thickness of about 1 μm, and the Si of the handle layer is of a thickness of 510 μm and resistivity of greater than 10000 Ω cm. A Si mesa with a height of 1.15 μm, and a width of 5–20 μm is defined by UV photolithography and ICP etching. Then use photolithography, dual ion beam sputtering, and a standard lift-off process to achieve metal Au (300 nm)/Pt (60 nm)/Ti (80 nm) on both sides of the mesa, forming a central gap of 5–15μm in the length direction of the mesa. The planar detector utilizes UV lithography, dual ion beam sputtering and a standard lift-off process to deposit metal directly on the device layer to produce SOI detectors with a channel length and width of 5 μm. The material and thickness of the gold electrode of the planar detector is the same as that of the mesa detector. Use a muffle furnace to anneal at 475°C for 1 h, so that metal-semiconductor Ohmic contacts are formed on both sides of the mesa. The two wires at the ends of the two metal electrodes are respectively connected to the preamplifier.

#### Characterizations and measurements

The photoelectric properties of Si detectors are tested at room temperature. Terahertz wave source is a backward wave oscillator (BWO 0.201–0.385 THz), the spot diameter is about 1cm. The power density at 0.23–0.32 THz is around 0.16 mW/cm^2^. The data of response time were acquired from an oscilloscope (Teledyne LeCroy 62Xi-A). The photovoltage signal was read out after the preamplifier (SR 570), the bandwidth of the preamplifier is 100 Hz-30 KHz and lock-in amplifier (SR 830).

### Quantification and statistical analysis

Origin 8 was used to generate the visual images in the manuscript. Experimental data was acquired using Labview.

## Data Availability

Data reported in this paper will be shared by the lead contact upon request. There is no dataset or code associated with this work. Silicon-on-insulator (SOI); electromagnetic induced wells (EIWs); sensitivity; terahertz detection
