# Supplementary material for: High sensitivity of room-temperature terahertz photodetector based on silicon
Source: iScience. 2022 Sep 26;25(10):105217. doi: 10.1016/j.isci.2022.105217 (PMC9557040; doi:10.1016/j.isci.2022.105217)
Supplement: Document S1. Figures S1–S7 [file mmc1.pdf]

iScience, Volume *25*

Supplemental information

High sensitivity of room-temperature terahertz photodetector based on silicon

Qinxi Qiu, Wanli Ma, Jingbo Li, Lin Jiang, Wangchen Mao, Xuehui Lu, Niangjuan Yao, Yi Shi, and Zhiming Huang

Supporting Information

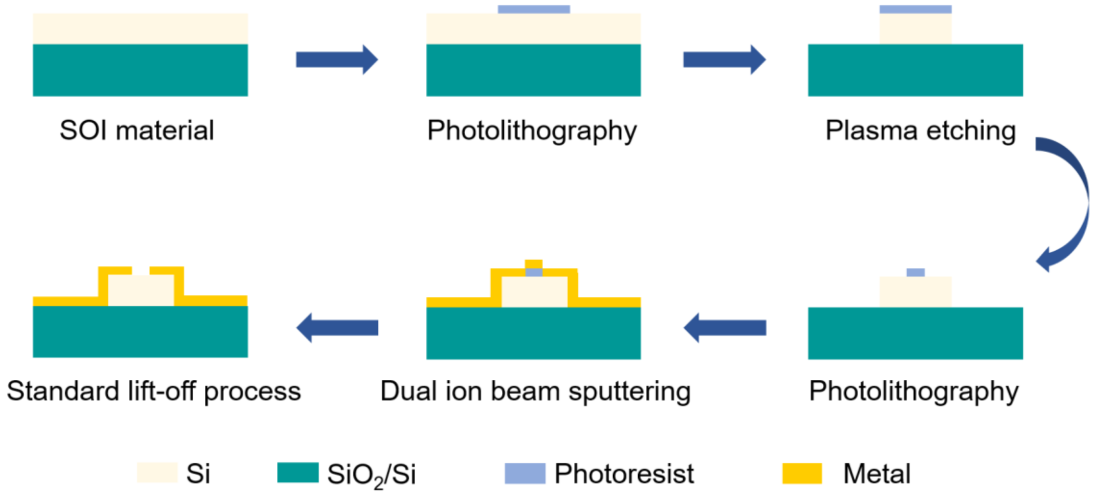

Figure S1. Fabrication process of SOI terahertz detector with a mesa. First, the mesa part of the SOI material is protected by the first photolithography, and the mesa is etched by plasma etching. Then the gap of the detector is protected by second photolithography, and metal is sputtered on the material by dual ion sputtering, and finally, the SOI THz detector with a mesa is fabricated using a standard lift-off process. Related to **Star Method**.

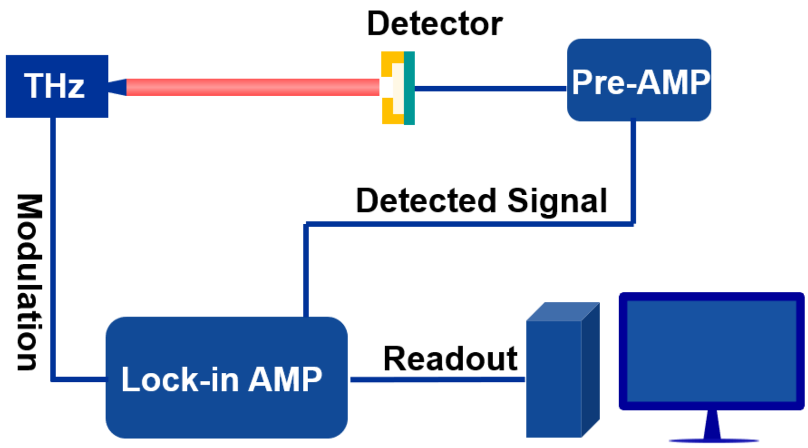

Figure S2. Schematic diagram of terahertz photoelectric response test. During the measurements, the distance between the detector and the incident source is 10 cm, the THz wave modulated by a square waveform at the frequency of 1 kHz is vertically incident to the surface of the detector. Related to **Figure 3**.

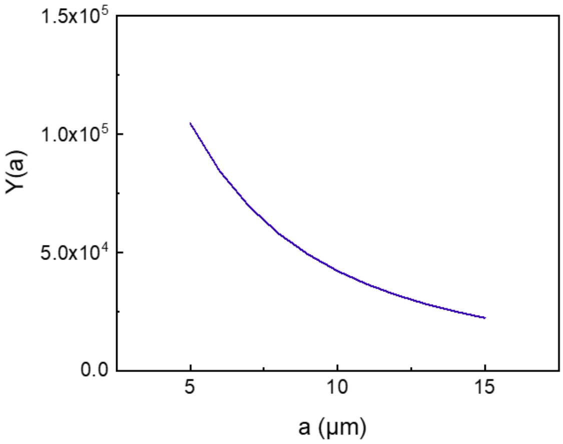

Figure S3. Plot of Y(a) versus a. Related to **Equation 4**.

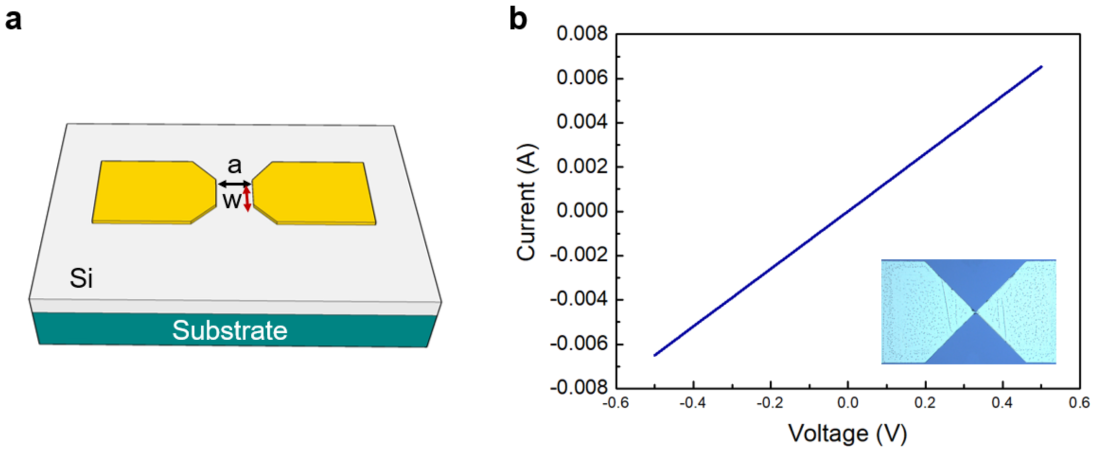

Figure S4. Schematic diagram of a planar Si detector and its I-V characteristics. a) Schematic diagram of the planar Si detector. b) Dark current I-V curve of planar SOI terahertz detector. Inset: Optical photograph of planar SOI terahertz detector. Related to **Figure 3a**.

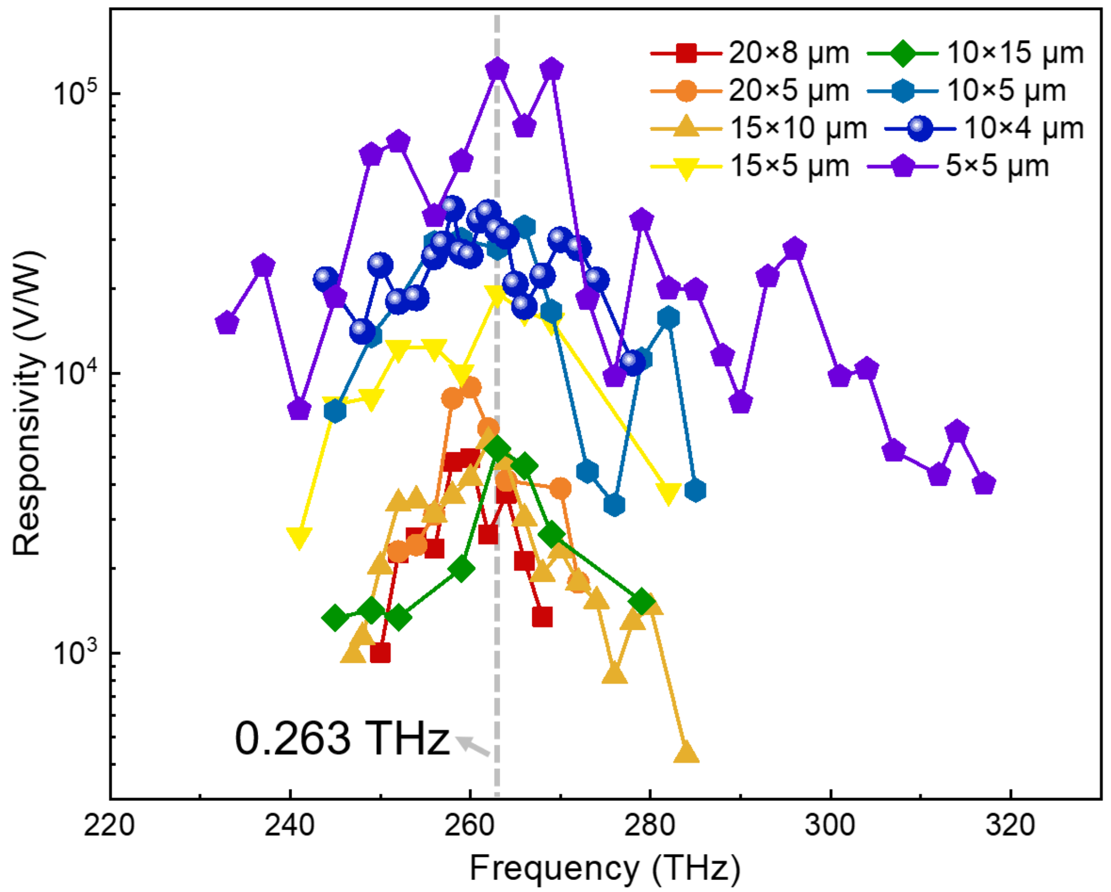

Figure S5. The responsivity of detectors with different mesa widths and gap lengths in 0.23-0.32 THz. Most detectors have the maximal responsivity at 0.263 THz, and the maximal responsivity of individual detectors is around 0.263 THz. Since the power density near 0.263 THz is 0.16-0.169 mW/cm^2, we select the maximal responsivity of the detectors for comparison. Related to **Figure 3b**.

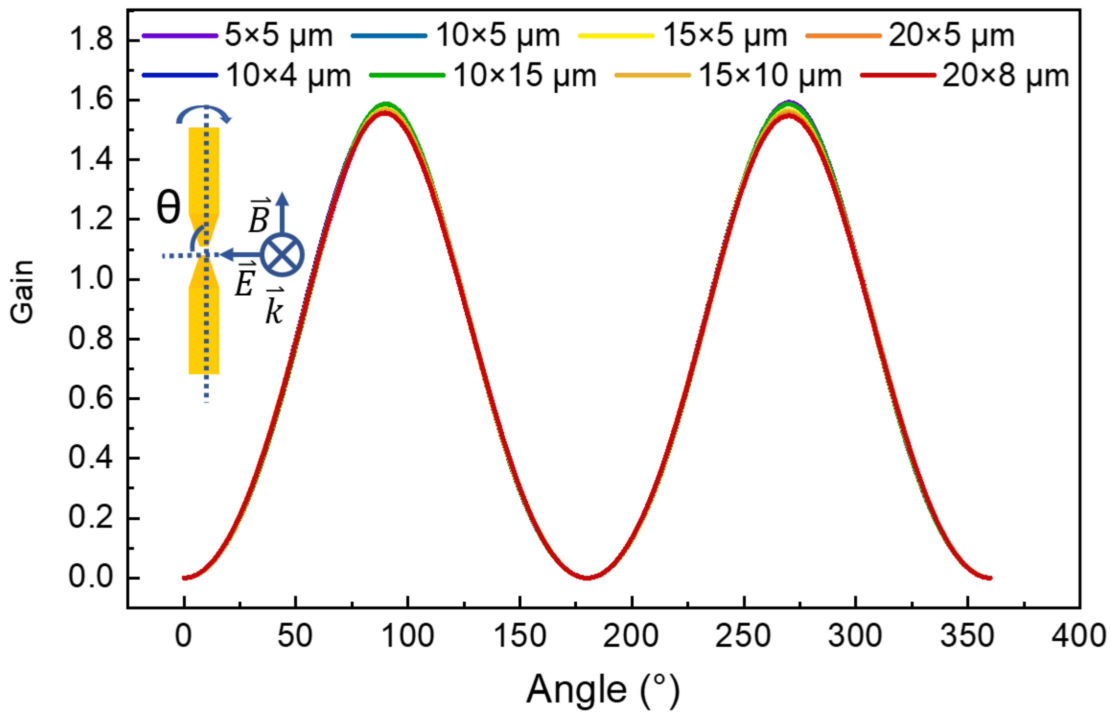

Figure S6. Numerical simulate the relationship between the antenna gain and the angle between the electric field of the terahertz wave and the antenna at 0.263 THz for detectors with different mesa widths and gap lengths. All detectors have the maximum gain at 90° and 270°, and the gain values of different detectors are not much different (1.556-1.587). Therefore, we will not consider the effect of antenna gain on the response of detectors of different sizes. Inset: schematic diagram of the angle between the electric field of the terahertz wave and the detector antenna. Related to **Figure 3b**.

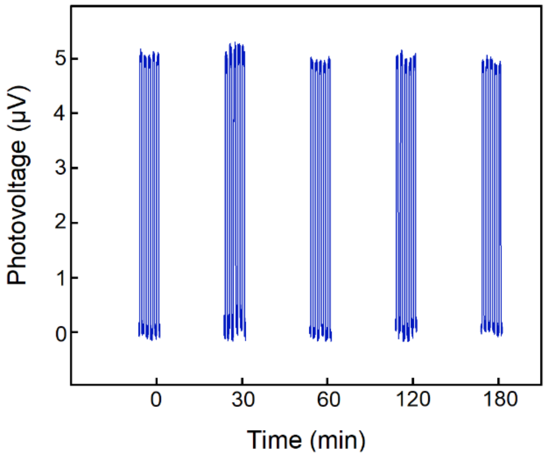

Figure S7. The photoresponse stability was measured on the Si detector under continuous irradiation at 0.263 THz at 5 mA. As shown in the figure, 97% of the initial light response remained good during 3 hours of exposure. Related to **Figure 4**.
